# The Role of Hydrogen-Peroxide (H_2_O_2_) Produced by Vaginal Microbiota in Female Reproductive Health

**DOI:** 10.3390/antiox12051055

**Published:** 2023-05-06

**Authors:** Eva Miko, Aliz Barakonyi

**Affiliations:** 1 Department of Medical Microbiology and Immunology, Medical School, University of Pécs, 12 Szigeti Street, 7624 Pécs, Hungary; barakonyi.aliz@pte.hu; 2 Janos Szentagothai Research Centre, 20 Ifjusag Street, 7624 Pécs, Hungary; 3 National Laboratory on Human Reproduction, University of Pécs, 7624 Pécs, Hungary

**Keywords:** vaginal microbiota, lactobacilli, hydrogen peroxide, probiotics, reproductive health

## Abstract

Female reproductive health is strongly associated with healthy vaginal microbiota, which is thought to be ensured by the dominance of certain *Lactobacillus* species. Lactobacilli control the vaginal microenvironment through several factors and mechanisms. One of them is their ability to produce hydrogen peroxide (H_2_O_2_). The role of *Lactobacillus*-derived H_2_O_2_ in the vaginal microbial community has been intensively investigated in several studies with many designs. However, results and data are controversial and challenging to interpret in vivo. Defining the underlying mechanisms responsible for a physiological vaginal ecosystem is crucial since it could directly affect probiotic treatment attempts. This review aims to summarize current knowledge on the topic, focusing on probiotic treatment possibilities.

## 1. Introduction

The unique vaginal microbial community with the dominance of *Lactobacillus* species, described by Döderlein more than a century ago, is considered the hallmark of the healthy vaginal microenvironment and the guarantee of overall vaginal health in women of reproductive age [1,2,3,4,5,6]. According to the predominant lactobacilli, vaginal microbiota composition is characterized by community state types (CSTs) [1]. Currently, CSTs are grouped in 6 bacterial configurations: CST I–*Lactobacillus crispatus*, CST II–*Lactobacillus gasseri*, CST III–*Lactobacillus iners*, CST V–*Lactobacillus jensenii* [1,7]. The CST IV lacks the significant abundance of a certain *Lactobacillus* species, and it can be divided into subgroups CST IV-A (modest proportion of *L. iners* and anaerobic bacteria: *Corynebacterium*, *Finegoldia*, *Streptococcus*, *Anaerococcus*) and CST IV-B (bacterial vaginosis associated bacteria: *Gardnerella*, *Atopobium*, *Prevotella* [7]. In approximately 90% of women of fertile age, the most prevalent configurations are CSTs I, III and IV [1].

An association between ethnicity and vaginal bacterial community composition was also demonstrated. CST IV (diverse group lacking *Lactobacillus* dominance) is significantly overrepresented and considered as common and normal in Hispanic and black women as compared with Asian and white women [1]. The reasons for the observed differences among ethnic groups are unknown but suggest host genetic factors determining vaginal bacterial colonization.

Changes and variations in community composition could be mainly affected during the menstrual cycle, by sexual activity or by other unknown factors. However, the vaginal microbiota of some individuals does not demonstrate temporal fluctuation and remains stable over several menstrual cycles [7]. In most cases, altered community composition did not affect community function since changes were observed in the relative abundance of a small number of different lactic acid-producing bacterial species [2,7].

During pregnancy, the diversity and richness of vaginal microbiota decrease compared to the non-pregnant vagina, with a higher abundance of *Lactobacillus* species [8,9,10]. Additionally, the stability of vaginal microbiota was significantly higher in the early stages of pregnancy and further increased with gestational age [11].

CST I with vaginal *L. crispatus* dominance is thought to be associated with a healthy vagina, while *L. iners*-dominance (CST III) is more prone to vaginal dysbiosis [12,13]. Multiple studies have shown the protective effect of *L. crispatus* against bacterial vaginosis (BV), vulvovaginal candidiasis (VVC), and sexually transmitted infections (STIs) [12,13].

There are several factors and mechanisms how lactobacilli contribute to maintaining a healthy vaginal ecosystem and preserving their dominance over other vaginal microorganisms (summarized in Table 1). It is most likely the combination of these lactobacilli-derived factors responsible for vaginal health and for the prevention of BV, VVC and STIs primarily [2].

To which extent each mechanism can contribute to a healthy vaginal mucosal microenvironment needs further investigation. For example, the production of lactate acid by vaginal lactobacilli and, thus, the reduced pH in the vagina directly inhibits the growth of a significant proportion of microorganisms occurring in or entering the vagina.

Since the main species of vaginal *Lactobacillus* can generate hydrogen peroxide (H_2_O_2_) and considering the chemical character of the molecule, H_2_O_2_ could be another critical element ensuring vaginal *Lactobacillus* abundance [14,15]. However, findings of clinical and experimental studies revealed inconsistent data and therefore, the significance of vaginal H_2_O_2_ is often doubted. This review aims to summarize current knowledge on the possible role of *Lactobacillus*-derived H_2_O_2_ in vaginal health.

## 2. Characteristics of Hydrogen Peroxide as a Chemical Compound and Oxidizing Agent

Hydrogen peroxide is a water-soluble liquid with an ashen blue color. Besides exogenous sources, such as microbial synthesis, which is the topic of this review, it can be produced in vivo as well, both enzymatically and non-enzymatically. H_2_O_2_ is generated by superoxide dismutase through the dismutation of the superoxide anion, or it can be produced directly by different oxidases in the human body (e.g., monoamine oxidase). Non-enzymatically formation of hydrogen peroxide occurs in the mitochondrial matrix as a result of the reduction of the superoxide anion by protons and electrons during terminal oxidation of the glucose metabolic pathway [16,17].

**Figure 1:**
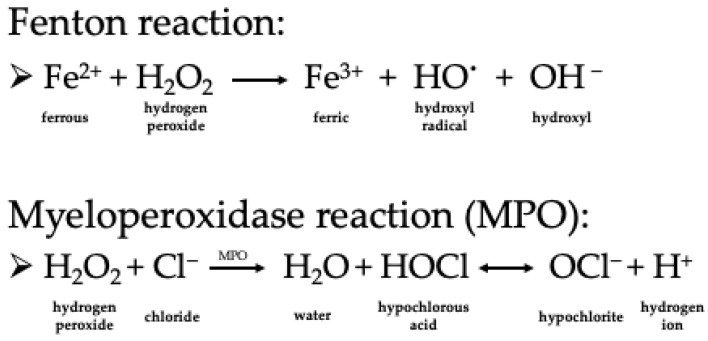
Conversion mechanisms of hydrogen peroxide in vivo.

Once present in human tissue, H_2_O_2_ can easily cross the cell membrane and act as an oxidizing agent in redox reactions. However, its oxidative capacity remains low, and it is instead an exception with its relatively stable character among reactive oxygen species occurring physiologically in humans [18]. The significant negative impact of hydrogen peroxide is its conversion into the dangerous hydroxyl radical, which rapidly oxidizes cellular macromolecules like DNA, proteins and lipids, acting thereby mutagen/carcinogen and/or direct cytotoxic. The Fenton reaction (soluble Fe(II) donates an electron to a hydrogen peroxide molecule, which splits then into hydroxyl anion and hydroxyl radical spontaneously) is thought to be responsible for the conversion in vivo, as shown in Figure 1 [17,19]. Moreover, H_2_O_2_ can release iron from the protein-bound form (heme proteins) and accelerate Fenton chemistry [17,20,21]. Another possible mechanism in humans is the enzyme myeloperoxidase (MPO), which in the presence of halides (like Cl^−^), is also able to convert H_2_O_2_ to hypochlorite (HOCl)(see Figure 1) [22]. It has been shown that cervicovaginal fluids contain adequate levels of MPO, and chloride is also present in the vaginal mucus [14,22].

Interestingly, the relative stability of hydrogen peroxide allows the molecule to exert essential and valuable functions for the organism before its spontaneous conversion into a harmful agent. In recent years, a growing body of evidence supported the role of hydrogen peroxide as a signaling molecule, more precisely, as a second messenger. After the formation of H_2_O_2_, there is an undefined period for the molecule to communicate the circumstances, namely the oxidative stress in its local environment, by binding to signaling proteins in the cascade [23,24,25,26,27,28]. One of the known targets is the important ubiquitous inflammation mediator, NFκB regulating cell proliferation, apoptosis and tissue repair [27,29,30,31].

## 3. Bacterial Hydrogen Peroxide Production

Besides endogenous sources of H_2_O_2_ in the human body, the occurrence of the molecule in the mucosa is mainly of microbial origin produced by local microbiota members. Lactic acid-producing bacteria, like lactobacilli, streptococci and pneumococci, can release detectable amounts of H_2_O_2_ in their environment. These bacteria lack heme and cytochrome proteins for terminal oxidation and utilize flavoproteins which synthesize H_2_O_2_ from O_2_ by oxidizing lactate, pyruvate or NADH with the corresponding enzymes [32,33,34,35]. Bacterial H_2_O_2_ can accumulate to a certain extent on mucous membranes since lactic acid bacteria cannot convert it with enzymes like catalase or peroxidase (e.g., NADH peroxidase) by themselves [14,15]. Hydrogen peroxide generating enzymes of bacterial origin were shown to be constitutively expressed, suggesting that H_2_O_2_ synthesis mainly depends on environmental O_2_ [36]

### 3.1. Hydrogen Peroxide Production in the Vaginal Microbiota

The healthy vaginal microbiota is dominated by the *Lactobacillus* genus. Although hydrogen peroxide formation by vaginal lactobacilli is a standard feature, it is an exception among intestinal and environmental strains [15]. Synthesis of H_2_O_2_ by vaginal lactobacilli is not species-specific but only characteristic of those with facultative anaerobe metabolism [37]. Vaginal colonization by H_2_O_2_-producing lactobacilli is as high as 10^6–^10^7^ organisms per ml [15]. Since different levels of H_2_O_2_ formation were shown for *L. crispatus*, there is a variation in hydrogen peroxide production between strains from the same species varying from excellent to non-H_2_O_2_ producers [38,39]. According to measurements of cultivated lactobacilli with different detection methods, amounts of H_2_O_2_ produced by the most active strains vary from 1.5–2 to 28–30 mg/L, and its detection is only possible after oxygenation of anaerobic cultures or under aerobic culture conditions, which is however, not characteristic for the vaginal lumen microenvironment [40,41,42]. Notably, the *L. iners* does not generate H_2_O_2_ [4,12].

The physiological hydrogen peroxide concentration of the cervicovaginal fluid is 23 ± 5 μM, which is considered low due to the relative oxygen shortage in the vaginal mucosa. Oxygen tension of [43,44] the vagina and consequentially enhanced H_2_O_2_ production by lactobacilli may increase, for example, during sexual intercourse, menstruation, inflammation and with the usage of intrauterine devices ([39,41]). Additionally, colonization by H_2_O_2_-producing *L. crispatus* or *L. jensenii* was more abundant in white women compared to other ethnical groups. [4].

In vitro studies demonstrated the cooperative actions of the primary metabolites of *Lactobacillus*, lactic acid and hydrogen peroxide. In the presence of lactic acid, H_2_O_2_ displayed enhanced antimicrobial killing activity against urogenital and enteric pathogens [42,45,46]. Since lactobacilli do not produce H_2_O_2_-inactivating enzymes (e.g., catalase) accumulation of hydrogen peroxide in the immediate area of the lactobacilli themselves may occur. This, in turn, may lead to autoinhibitory effects in lactobacilli caused by self-produced H_2_O_2_ [40].

### 3.2. Effects of Lactobacillus-Derived H_2_O_2_ on the Host

In the context of the vaginal microenvironment, locally produced H_2_O_2_ by lactobacilli may interact not only with other microorganisms but with vaginal mucosa and its components like epithelial and immune cells. Harmful effects of H_2_O_2_ as an oxidizing agent may occur in the host tissue. However, due to unpreventable endogenous H_2_O_2_ formations in eukaryotic cells, they are best equipped with neutralizing enzymes, e.g., catalase and peroxidase. As a part of the mucosa-associated lymphatic tissue (MALT), the vaginal mucosa harbors macrophages, natural killer (NK) cells, dendritic cells (DC) and neutrophils in variable distribution influenced mainly by hormonal changes [47,48,49]. The most abundant leukocytes are T lymphocytes, but γδ and mucosal-associated invariant T (MAIT) cells are also present in the vaginal mucosa [50,51,52,53]. IgG and IgA are also found in the vagina; interestingly, in cervicovaginal fluids, IgG concentration is higher than IgA [54]. B cells are found mainly in the ectocervix [53,55].

Possible immunomodulatory effects of H_2_O_2_ were suggested in a population-based study: cytokine concentrations were determined in cervicovaginal fluids revealing lower levels of vaginal interleukin (IL)-1β by the dominance of H_2_O_2_-producing lactobacilli [56]. IL-1β is a crucial mediator of inflammatory responses. It is released upon intracellular inflammasome activation, mainly by macrophages and dendritic cells. An innate immune subversion through inflammasome inhibition was demonstrated in a recent study showing that the oral commensal H_2_O_2_-producing bacterium *Streptococcus oralis* can block inflammasome activation, which is mediated by hydrogen peroxide [57].

In eukaryotes, hydrogen peroxide has important roles as a signaling molecule regulating various biological processes, like cell proliferation, differentiation, migration, or apoptosis [58]. Although hydrogen peroxide can diffuse across membranes, exogenously produced H_2_O_2_ is less effective at eliciting a signaling response than endogenously produced hydrogen peroxide [59]. The concentration of exogenous hydrogen peroxide sufficient to act as a second messenger varies significantly between cells [59,60]. Furthermore, different concentration-specific responses can occur in eukaryotic cells [61]. A large group of redox-regulated proteins is found in almost all cell types, including transcription factors, kinases, phosphatases, and ion channels [58]. A probiotic strain of *L. crispatus* M247 uses H_2_O_2_ as a signal-transducing molecule to induce PPAR-γ (peroxisome proliferator-activated receptors) activation in IEC, directly modulating epithelial cell responsiveness to inflammatory stimuli. PPAR-γ is an endogenous regulator of intestinal inflammation; its activation prevents inflammatory damage in colitis [62].

**Figure 2:**
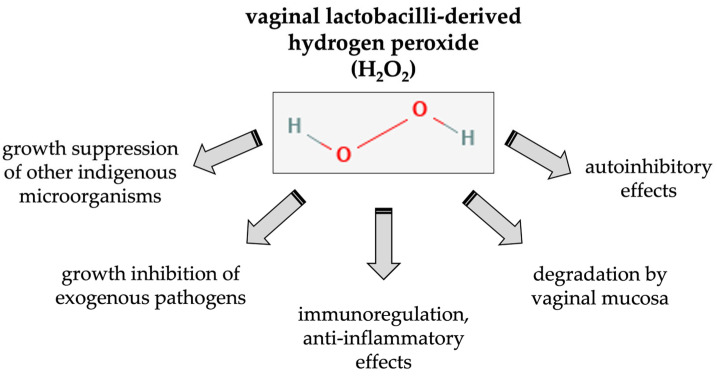
Suggested effects of *Lactobacillus*-derived H_2_O_2_ in the vaginal mucosa and microbial community.

Figure 2 summarizes the possible main effects of *Lactobacillus*-derived H_2_O_2_ in the vaginal mucosa and microbial community.

## 4. The Possible Contribution of *Lactobacillus*-Derived Hydrogen Peroxide to Vaginal Health: Pros and Cons

In the vaginal micro-ecosystem, the primary impact of hydrogen peroxide on living organisms at the cellular level is believed to be predominantly antimicrobial. The control of the growth of specific microbial populations could contribute to the physiological composition of the vaginal microbiota and ensure the dominance of H_2_O_2_-producing lactobacilli. There are mainly two types of studies investigating the effects of vaginal H_2_O_2_. Epidemiological studies focused on the presence and ratio of H_2_O_2_-producing lactobacilli and its possible association with vaginal dysbiosis and/or infection. Experimental studies were of the microbiological type, investigating characteristics and requirements of bacterial H_2_O_2_ synthesis in the culture of vagina-derived *Lactobacillus* species and its effect on other microbial populations. While epidemiological investigations rather support the protective role of hydrogen peroxide-producing lactobacilli in vaginal health, many in vitro studies failed to demonstrate a significant role of H_2_O_2_ in maintaining physiological vaginal microbiota composition.

### 4.1. Antimicrobial Effects of H_2_O_2_: Epidemiological Studies on Bacterial Vaginosis

The first studies focusing on this topic were in vivo observational studies beginning from the late 1980s. Their provide the most supporting data about the positive correlation between the vaginal presence and dominance of H_2_O_2_-producing *Lactobacillus* species and healthy vaginal microbiota. One group of these studies focused on the association between H_2_O_2_ lactobacilli and bacterial vaginosis. BV is thought to be a vaginal dysbacteriosis with anaerobic overgrowth (e.g., *Gardnerella*, *Atopobium*) with or without symptoms [63,64]. Women with bacterial vaginosis have higher risks for preterm birth, late miscarriage, and HIV infection [65,66,67].

The epidemiological studies on bacterial vaginosis demonstrated a remarkable difference in the prevalence of isolated H_2_O_2_-producing lactobacilli in healthy, nonpregnant women and women with bacterial vaginosis. Hydrogen peroxide-generating lactobacilli were detected in the large majority of healthy women whereas only in a small part of women with BV [15,68]. Moreover, differences in vaginal colonization by lactobacilli in healthy women and those with BV were only observed in the H_2_O_2_-producing group of *Lactobacillus* [68,69]. Since the absence of these bacteria in women with bacterial vaginosis was more prevalent than the increased colonization rates of anaerobic bacteria (*Gardnerella*, *Mobiluncus*, Mycoplasma), the theory of the presence of H_2_O_2_ producing lactobacilli as a critical protective factor in the healthy vaginal microbiota was reasonable [15].

These findings also raised an important question regarding the pathogenesis of BV and the chronology of microbial events resulting in the disease. Do H_2_O_2_-positive *Lactobacillus* species first disappear, and do obligate anaerobic bacteria take over their place or the other way round? A possible answer was provided by longitudinal studies of healthy, nonpregnant women with follow-up visits. They confirmed the lack of H_2_O_2_-forming lactobacilli as a primary risk factor for bacterial vaginosis [70]. BV development was four times higher in women without Lactobacillus species producing H_2_O_2_ than in women colonized by these bacteria. Harboring any lactobacilli reduced the risk of BV twofold [70]. The acquisition of bacterial vaginosis was significantly higher among women initially colonized with H_2_O_2_-producing strains and lost colonization of these species later, compared to persistently colonized women [71]. H_2_O_2_-positive strains of *L. crispatus* and *L. jensenii* were the most likely to maintain persistent vaginal colonization over the period of the study, suggesting optimized host-microbiota interactions.

Interestingly, vaginal and rectal co-colonization by H_2_O_2_-producing *L. crispatus* species is suggested as another protective factor against BV development. In a cross-sectional study, co-colonization by H_2_O_2_-positive lactobacilli was shown to occur very often and was associated with a reduced risk of BV 4-fold compared with vaginal colonization only [72]. According to this observation, H_2_O_2_-producing lactobacilli in the distal gastrointestinal tract could contribute to maintaining the dominance and supplying eventual shortages of vaginal lactobacilli.

Epidemiological studies also helped identify several demographic and behavioral factors correlated either positively or negatively with vaginal colonization of H_2_O_2_-producing *Lactobacillus* species. Certainly, several of these factors are also correlated to the development of bacterial vaginosis. Vaginal colonization with H_2_O_2_-forming *Lactobacillus* strains was associated with white race, higher education, the use of barrier contraception and less smoking [70]. Older age, parity, alcohol use, having ≥1 act of vaginal intercourse per week, vaginal cleansing, current BV, and recent use of antibiotics were associated with decreased H_2_O_2_+ *Lactobacillus* isolation. women having ≥1 act of vaginal intercourse per week (no information about condom use) or antibiotic treatment were more likely to lose colonization [71,73]. The possible association with host-specific health issues (e.g., hormone and immunologic status) has not been investigated.

When determining the levels of H_2_O_2_ in vaginal secretions, women with BV had lower levels of H_2_O_2_ than healthy women’s concentrations (0.04 μg/mL vs. 0.17 μg/mL) [74].

The hypothesis of the protective role of H_2_O_2_-producing lactobacilli in the vagina was questioned by some studies suggesting that bacterial vaginosis may develop despite the presence of lactobacilli with H_2_O_2_ formation [75,76]. For example, in most of the investigated BV cases with large numbers of BV-associated species, simultaneous colonization of vaginal lactobacilli in large numbers (10^5^–10^6^ colony forming units (CFU)/mL) was demonstrated. Moreover, as shown in vitro, strong H_2_O_2_ producers were identified in BV cases as well [75,76]. However, due to the more significant number of lactobacilli observed in healthy women, it might be the case that overall higher amounts of vaginal H_2_O_2_ would be generated in them than in women with bacterial vaginosis [76,77].

### 4.2. Antimicrobial Effects of H_2_O_2_: Epidemiological Studies on Vulvovaginal Candidiasis (VVC)

One of the most frequent vaginal disorders is vulvovaginal candidiasis, caused by several species of the yeast *Candida*, predominantly by *C. albicans*. *Candidal* vulvovaginitis occurs when *Candida* species members of vaginal microbiota superficially penetrate the mucosal lining of the vagina leading to a secondary inflammatory response [78]. An association between *Candida* overgrowth and levels of lactobacilli overall, neither a deficiency nor colonization with unusual *Lactobacillus* species could be observed [79]. Most women with candidiasis had the highest lactobacilli counts; even previous antibiotic treatment did not affect lactobacilli density [80]. Similar results were shown later: hydrogen peroxide-producing *Lactobacillus* species were almost equally isolated in women with normal microbiota and women with VVC [80]. Furthermore, a longitudinal study showed no correlation between initial H_2_O_2_−/H_2_O_2_+ *Lactobacillus* colonization with the development of symptomatic candidiasis. The demonstration of the protective role of H_2_O_2_-positive lactobacilli against the acquisition of VVC failed and therefore a possible correlation was questioned [37]. Vaginal *Candida* propagation may be facilitated by fungal intrinsic and/or local extrinsic factors more powerful than *Lactobacillus*-related defense.

### 4.3. Antimicrobial Effects of H_2_O_2_: Epidemiological Studies on STI Pathogens

Hydrogen peroxide produced by lactobacilli in the vagina may not only maintain their dominance and control the physiological composition of the vaginal microbiota, but they may protect against colonization of pathogens. Preventing sexually transmitted diseases and ascending infection of the chorioamniotic membranes and uterine cavity in pregnant women is of great medical importance. Compared to in vitro studies, there are just a few investigations dealing with epidemiologic correlation between H_2_O_2_-producing lactobacilli and vaginal infection [37,70,80,81,82]. Most of these epidemiological studies are complex, analyzing normal microbiota, bacterial vaginosis, and the most frequent infections simultaneously.

Infections by the protozoon *Trichomonas vaginalis* belong to the group of sexually transmitted diseases, with having a new sex partner as the most important risk factor. Since *T. vaginalis* is able to the phagocytosis and indirect killing of vaginal lactobacilli with its toxic metabolic products, abnormal vaginal flora and/or reduction of lactobacilli are thought to be additional risk factors for the infection [80,83,84]. Most epidemiological studies investigating the possible role of H_2_O_2_-producing *Lactobacillus* species in normal vaginal flora, bacterial vaginosis and VVC failed to show any correlation of the bacteria with trichomoniasis [37,70,80].

In the case of the STI caused by *Neisseria gonorrhoeae*, *Lactobacillus*-dominant vaginal microbial community was shown to protect individuals from lower genital tract infection with *N. gonorrhoeae* [85]. Furthermore, women colonized by H_2_O_2_-generating *Lactobacillus* species were less frequently infected by gonococci than women lacking H_2_O_2_+ lactobacilli [70].

The association between HIV infections and vaginal colonization by H_2_O_2_-forming lactobacilli was investigated in HIV seronegative and seropositive women. Compared with African female sexual workers carrying H_2_O_2_+ *Lactobacillus*, women without lactobacilli had a 2.5-fold higher risk of HIV-1 infection. Women with only H_2_O_2_-negative strains were at intermediate risk. The abundance of H_2_O_2_-generating lactobacilli in HIV-positive women was significantly reduced than in HIV-negative women [81]. Analyzing the occurrence of different hydrogen peroxide-producing *Lactobacillus* species in HIV seropositive women, it was demonstrated that H_2_O_2_-producing *L. gasseri* is more prevalent in the population of HIV-1 infected women [82]. Moreover, it was the predominant species detected among women who had high quantities of H_2_O_2_-producing *Lactobacillus* but were negative for both *L. crispatus* and *L. jensenii*, suggesting an alteration of *Lactobacillus* species in the vaginal flora of HIV-positive women [82].

### 4.4. Antimicrobial Effects of H_2_O_2_: Experimental Studies

Alongside the population-based studies, the hypothesized direct antimicrobial role of vaginal lactobacilli-derived hydrogen peroxide was investigated in a series of experiments. In these microbiological in vitro studies, the H_2_O_2_-mediated killing/inhibition of target pathogens was observed by co-culturing them either with different types of lactobacilli or with its supernatants or with cervicovaginal fluid.

The most convincing epidemiological correlation was demonstrated in women with bacterial vaginosis, where vaginal colonization by H_2_O_2_-producing *Lactobacillus* strains was reduced. The key bacterium of BV is thought to be *Gardnerella vaginalis*, with its overgrowth potential beside certain other anaerobes. Initial findings of in vivo studies assumed the control of H_2_O_2_-forming *Lactobacillus* species over other members of the vaginal microbiota, especially over *G. vaginalis* with the, at least partially, direct bactericidal effect of H_2_O_2_.

In a liquid co-culture assay with lactobacilli and BV-associated organisms, *G. vaginalis* and *P. bivia* at pH 5 resembling vaginal acidic conditions, killing of the pathogens could be observed only when H_2_O_2_-producing lactobacilli were added to the system, H_2_O_2_-negative lactobacilli showed no effect [14]. The H_2_O_2_-dependent antibacterial mechanism was demonstrated by adding catalase to the assay, which abandoned the reduction of *G. vaginalis* and *P. bivia*. Moreover, toxic effects on *G. vaginalis* could be augmented by adding peroxidase and a halide to the co-culture [14]. However, the study has some limitations in its interpretation in vivo. First, as shown in studies later, reduced pH alone can inhibit the growth of several vaginal microbiota members, including *G. vaginalis* [86]. Therefore, bacterial depletion should have also occurred in the test system with H_2_O_2_-nonproducer lactobacilli. Furthermore, experiments were carried out under fully aerobic conditions, which is questionable in the vaginal mucosa. At toxic concentrations of H_2_O_2_ complemented with peroxidase and a halide in the experiments, there was a fall in the viable cell count of the H_2_O_2_-producing lactobacilli, suggesting autoinhibitory effects. Since lactobacilli dominate the vaginal microbiota, this suggests more reduced H_2_O_2_ concentrations in vivo and a rather subordinate role of oxidative stress caused by H_2_O_2_ in controlling the growth of BV-associated bacteria.

Another study of the same year with co-culture test systems, although on solid agar media, supported these concerns regarding in vitro experiments [86]. 5 of 20 H_2_O_2_-positive lactobacilli isolated from healthy women or women with bacterial vaginosis exerted some inhibitory effects against a few *Mobiluncus* and *Peptostreptococcus* strains. In contrast, others failed to reduce the growth of *Gardnerella vaginalis*, *Bacteroides* spp. and other strains of *Mobiluncus* and *Peptostreptococcus*. Similar results were observed with H_2_O_2_-negative lactobacilli. The observed antimicrobial effect was hardly influenced by the pH of the medium, suggesting rather pH-dependent growth inhibition. A growth inhibitory activity of H_2_O_2_ alone at different concentrations (0.0003–0.3%) was not observed either. It should be noted that these experiments were carried out under anaerobic conditions, and concentrations of target bacteria were chosen arbitrarily and, therefore, probably too high for inhibition detection (10^6^ CFU/mL) [63].

A more complex study provided detailed data about the circumstances of H_2_O_2_ production and H_2_O_2_-mediated toxicity by vaginal lactobacilli [87]. In these experiments, the inhibitory effects of 22 isolated H_2_O_2_-producing vaginal lactobacilli were determined on different *G. vaginalis* strains. Agar well diffusion assay measured the cell growth reduction induced by *Lactobacillus* culture filtrates. The influence of several culture parameters (pH, H_2_O_2_ presence, anaerobic/aerobic conditions) was tested independently. A low pH of around four and lactic acid accounted for 60 to 95% *Lactobacillus*-derived inhibitory activity, and H_2_O_2_ accounted for only 0 to 30% after its denaturation with catalase treatment. H_2_O_2_ production was not detectable under anaerobic or static aerobic conditions. This study also confirmed the enhancement of killing with additional peroxidase and halide under aerobic conditions. These findings suggest that lactic acid and a low pH are more critical for *G. vaginalis* growth inhibition than H_2_O_2_ in vitro [87].

Comparison of the antimicrobial effect of pure H_2_O_2_ and culture supernatants of H_2_O_2-_producing vaginal lactobacilli against BV-associated microorganisms demonstrated significant H_2_O_2_ sensitivity of *Gardnerella* and *Prevotella*, however, experiment conditions are unlikely to occur in vivo [39]. Catalase treatment neutralized growth inhibition of pure H_2_O_2_ but did not affect the toxicity of culture supernatants proposing other toxic mechanisms than H_2_O_2_. Cultured media of *Lactobacillus* species with moderate or low H_2_O_2_ production appeared to be less effective or ineffective on the growth of G. vaginalis, indicating dose-dependent toxicity and varying levels of H_2_O_2_ and at least in vitro [38].

In the case of candidiasis, as seen in the epidemiological studies, the protective role of lactobacilli-derived hydrogen peroxide is rather doubtful since different *Candida* species were found to be resistant to relatively high concentrations of H_2_O_2_ [40]. 30 g/L H_2_O_2_ was necessary to kill all *Candida* yeast cells, and 3 g/L was inhibitory for only some *Candida* cells [40]. No *Lactobacillus* strain was found to produce H_2_O_2_ in this high concentration. Moreover, this concentration of H_2_O_2_ would also act as an autoinhibitory [40]. In contrast to these results, using *Lactobacillus* culture supernatants, in which H_2_O_2_ reached concentrations from 0.05 to 1.0 mM, they effectively could inhibit *Candida* growth. Still, it could not be neutralized with catalase, suggesting other toxic mechanisms [39]. Furthermore, *Candida* species can produce their catalase for H_2_O_2_ degradation, which, in turn, could be further stimulated by *Lactobacillus*-derived H_2_O_2_ [39]. In another study of the same year, the minimal bactericidal concentration of H_2_O_2_ on a single *C. albicans* strain was 2.52 mmol/L; *Candida albicans* appeared to be approximately six times more tolerant than *G. vaginalis* to H_2_O_2_-mediated inhibition [38]. Supernatants of vaginal H_2_O_2_-producing lactobacilli, treated with proteinase K to neutralize antimicrobial peptides but with maintained H_2_O_2_ activity, showed insufficient eradication of *C. albicans* [38].

Liquid co-culture experiments demonstrated pH-dependent growth inhibitory effects of vaginal lactobacilli on the pathogen *Neisseria gonorrhoeae* with the enhancement of toxicity under acidic conditions [88]. Gonococcal growth was significantly more inhibited by H_2_O_2_-producing lactobacilli. Moreover, H_2_O_2_-positive lactobacilli could increase catalase production by *N. gonorrhoeae* at least at neutral pH, and catalase activity parallel decreased with pH, probably due to the bactericide effect of low pH on gonococci [88]. Co-culture experiments obtained similar results based on the sandwich method with agar plates investigating the inhibitory effect of four isolated *Lactobacillus* strains (*L. crispatus*, *L. jensenii*, *L. gasseri*, *L. acidophilus*) on two Gonococcal laboratory strains [42]. All four *Lactobacillus* strains inhibited the growth of all Gonococcal strains tested at low pH. Since adding catalase could effectively neutralize *Lactobacillus*-mediated Gonococcal killing, H_2_O_2_ was suggested as the primary mediator of inhibition [42].

In one study, supernatants of H_2_O_2_-producing *Lactobacillus* species were shown to inactivate elementary bodies of *Chlamydia trachomatis* mainly through a lactate acid-dependent mechanism since catalase-treatment could not reverse the inhibition by neutralizing H_2_O_2_ [43]. It was hypothesized that the rigid outer membrane of Chlamydia could prevent H_2_O_2_ from entering the cell [43].

The antiviral potential of H_2_O_2_ was studied for Herpes Simplex Virus Type 2 (HSV-2) and Human Immunodeficiency Virus Type 1 (HIV-1) [89,90]. Inhibition of HSV-2 multiplication by lactobacilli was demonstrated on HSV-2-infected Vero cells incubated with bacteria. However, culture supernatants of H_2_O_2_-forming lactobacilli could neither modify the infectivity of HSV-2 virions nor affect intracellular events of virus multiplication. Investigation of the effects of pure H_2_O_2_ in cell culture experiments was hindered by the fact that H_2_O_2_ was metabolized promptly in the cell culture, and maintaining constant levels of H_2_O_2_ was not possible. Incubating HSV-2 virions with H_2_O_2_, hydrogen peroxide showed a dose-dependent reduction of the infection capacity with a 50% inhibition at 184 μM after one h incubation. Still, that high activity level in the vagina is rather unlikely [43,44,89]. In a simple, early study on HIV-1, H_2_O_2_-producing *L. acidophilus* at a concentration of 10^7^ CFU/mL was viricidal to HIV-1 virions in lactate buffer with pH 5.0 [90]. The role of H_2_O_2_ was demonstrated with the addition of myeloperoxidase and chloride to lower concentrations of the *Lactobacillus* with ineffective viricidal activity alone, showing enhancement of viral reduction in the in vitro test system [90].

The correlation of these studies with physiological conditions in the vaginal mucosa is challenging to estimate since in vitro studies contain several limiting factors for their proper interpretation. Most of the studies worked with isolated *Lactobacillus* strains and not with bacterial communities; concentrations of lactobacilli and target bacteria varied in the experiments, as was the case of O_2_ tension and sometimes pH values during incubation periods. Using pure H_2_O_2_ in inhibitory experiments, it should always be remembered that lactobacilli are H_2_O_2_-sensitive as well. Furthermore, cervical mucosa may harbor additional molecules able to react with and inactivate hydrogen peroxide. Using cervicovaginal fluid (CVF) from healthy women as the natural source of lactobacilli-derived compounds in in vitro experiments could eliminate some significant issues regarding microbiological tests [91,92]. Under hypoxic conditions, CVF lost its H_2_O_2_ content within one hour, significantly suggesting inactivating mechanism present in CVF [91]. The mean hydrogen peroxide content in CVF samples after aeration was only 23 ± 5 μM, one hundred times lower than maximal aerobic in vitro production (~2 mM). But even 50 μM hydrogen peroxide could not contain inactive pathogens like HSV-2, *N. gonorrhoeae*, *H. ducreyii* and several BV-associated bacteria. Additionally, adding 1% CVF reversed the in vitro inactivation of *G. vaginalis* and *P. bivia* by H_2_O_2_-producing *L. crispatus*, suggesting vigorous H_2_O_2_-blocking activity of CVF and questioning the dominant role of hydrogen peroxide in the maintenance of healthy vaginal microenvironment [91]. In contrast, a strong inhibitory effect was observed with physiological concentrations of lactic acid (56 mM) at pH 4.5 [92]. Although the advantage of examining CVF by the interpretation of the results, limitations of the experiments still exist. Localization, distribution, and stability of H_2_O_2_ in the vaginal mucosa and bacterial interactions in proximity in the tissue are still unknown characteristics of H_2_O_2_-producing lactobacilli in the vaginal microbiota.

### 4.5. Impact of H_2_O_2_-Producing Lactobacilli on Fertility and Pregnancy Outcome

In healthy pregnancy, the abundance of vaginal H_2_O_2_-producing lactobacilli was lower than that in nonpregnant females [93]. The proportion of H_2_O_2_+ strains decreased with the gravidae’s age and increased with pregnancy trimesters [93].

Several studies demonstrated the association of vaginal dysbiosis with the negative outcome of fertility treatments suggesting altered vaginal microbiota and bacterial vaginosis as independent risk factors with a predictive value [94,95,96,97,98]. Moreover, investigating 135 vaginal *Lactobacillus* strains belonging to the species *L. crispatus*, *L. jensenii* and *L. gasseri*, lactobacilli strains from healthy women generated significantly higher amounts of H_2_O_2_ than strains of infertile women [99]. Colonization with *Lactobacillus* species that produce hydrogen peroxide (H_2_O_2_) and bacterial vaginosis have been associated with lower rates of preterm birth as well, suggesting the protective role of these strains against ascending infections [100]. Furthermore, the presence of H_2_O_2_-producing species (*L. jensenii* and/or *L. vaginalis*) during pregnancy was associated with significantly reduced rates of preterm birth and/or chorioamnionitis [101].

## 5. H_2_O_2_-Producing Lactobacilli as Therapeutic Agents in Vaginal Probiotics

Considering the *Lactobacillus* dominance of the healthy vaginal microflora, efforts to modulate the pathological composition of vaginal microbiota with probiotics have been made in the last decades in varying studies [2,64]. Most of these trials focused on treating the most common bacterial vaginosis with its dysbiotic character, and a few studies focused on vulvovaginal candidiasis [102,103]. Probiotic treatment can be either primary, in combination, or following antibiotic therapy, or probiotics can be administered orally or vaginally.

Interestingly, the effects of vaginal douching with 3% H_2_O_2_ alone for treating BV have been investigated in two studies with contradictory findings [104]. In recurrent cases of bacterial vaginosis, daily vaginal irrigations with 30 mL of 3% hydrogen peroxide for seven days could eliminate the main symptoms of BV three months after treatment. This result was comparable to that achieved with local antibiotic therapy. Moreover, H_2_O_2_ was found to facilitate the restoration of normal vaginal bacterial flora and normal acid pH in 98% of cases and led to the disappearance of clue cells from vaginal smears [104]. In a randomized controlled trial, 3% H_2_O_2_ single vaginal douching was significantly less effective than a single oral dose of metronidazole (62.5% versus 78.6%) in treating bacterial vaginosis [105].

In studies aiming to characterize and select vaginal *Lactobacillus* species with probiotic potential, one of the primary criteria (e.g., lactic acid formation, epithelial adhesion, inhibitory activity against BV-associated bacterial species) is the H_2_O_2_-producing capacity of lactobacilli tested in vitro. Certain strains of several *Lactobacillus* species (*L. crispatus*, *L. acidophilus*, *L. jenseni*, *L. gasseri*, *L. brevis*, *L. salivarius*) were considered probiotic candidates based partly on strong H_2_O_2_ generation [99,106,107,108,109].

Local administration of an H_2_O_2_-producing probiotic strain of *L. crispatus* in healthy, sexually active women could establish *Lactobacillus* colonization in women lacking lactobacilli [110]. Furthermore, the introduction of exogenous *L. crispatus* increased vaginal colonization by other H_2_O_2_-producing lactobacilli in female participants. Failure of colonization with the probiotic strain was associated with sexual intercourse [110].

One of the first studies using probiotic strains (H_2_O_2_-producing *L. acidophilus*) in vaginal capsules (for the treatment of BV could not demonstrate any efficacy for the treatment of BV probably because of losing a significant proportion of patients during the trial [111,112]. In a multicentric, randomized, placebo-controlled clinical trial, locally administered estradiol-combined H_2_O_2_-producing *L. acidophilus* achieved significantly higher cure rate in non-menopausal women with BV [112]. Further trials showed varying results [113,114].

Combination therapies with different, mostly H_2_O_2_ *Lactobacillus* species revealed controversial results as well [115,116,117].

Interestingly, the high efficiency of oral probiotic therapy of bacterial vaginosis was demonstrated in clinical trials using well-characterized intestinal *Lactobacillus* strains: *L. rhamnosus* GR-1 and *L. reuteri* RC-14 either alone or in combination with antibiotic treatment. Vaginal *Lactobacillus* recovery suggests the ability of *Lactobacillus* GR-1 and RC-14 to colonize the vagina after oral intake [108,118,119,120]. Vaginal administration of these intestinal probiotic strains combined with antibiotic treatment demonstrated high efficiency in curing vaginal BV and stabilizing the vaginal ecosystem in a few studies [121,122,123,124,125]. Additionally, oral probiotic *L. reuteri* RC-14 and *L. rhamnosus* GR-1 strains increased the relative abundance of indigenous vaginal lactobacilli, like H_2_O_2_-producing *L. crispatus* [126].

Some trials have also investigated the efficiency of combination therapy of vulvovaginal candidiasis with antifungals and probiotics compared to antifungals alone, but no information was available about the H_2_O_2_ production of the administered probiotic strains in the studies [108,127,128,129,130,131,132].

Despite the strong negative association between vaginal dysbiosis and IVF outcome, probiotic therapy (*L. acidophilus* and bifidobacterial; H_2_O_2_ production not known) of infertile women was carried out only in two studies reporting treatment failure [133,134,135].

## 6. Conclusions

Several species of the genus *Lactobacillus* constitute a significant component of the human microbiota at various body sites, like the oral, intestinal, and vaginal flora. However, the abundance of hydrogen peroxide-producing lactobacilli is unique for the vaginal microbial community suggesting the importance of *Lactobacillus*-derived exogenous H_2_O_2_ in the vaginal microenvironment. Most epidemiological studies could support this theory and reveal the protective role of H_2_O_2_ against dysbiosis and pathogen colonization. In vitro studies contradicted these findings and demonstrated other possible effects of H_2_O_2_. Since these microbiological experiments were conducted under artificial circumstances with several arbitrary settings, it became apparent that interpreting the results referring to in vivo conditions is complicated and nearly impossible. Although in studies, aiming at the selection of vaginal *Lactobacillus* species with probiotic potential, selection criteria included the ability of the strain to produce H_2_O_2_, this was often not in the focus of the clinical trials with probiotic lactobacilli and the administration of many different *Lactobacillus* species and strains made challenging to draw valuable conclusions. However, going through all the studies with many aspects and designs, *Lactobacillus*-derived H_2_O_2_ still seems important in vaginal health. H_2_O_2_ production may play an antimicrobial role in the vaginal microenvironment, probably limited to local cell-cell interactions in close proximity, embedded in a biofilm that is not reproducible in vitro. Furthermore, H_2_O_2_-producing lactobacilli may possess a favorable, more efficient phenotype than their non-producer counterparts contributing significantly to a healthy vaginal ecosystem. Since altered vaginal microbiota is strongly associated with disorders of infective character (BV, VVC, STIs) and certain pregnancy-related complications (infertility, pre-term birth), further investigations are needed to explore the possible background and to improve probiotic treatment.

## Figures and Tables

**Table 1:** Contribution of lactobacilli to a healthy vaginal microbiota.

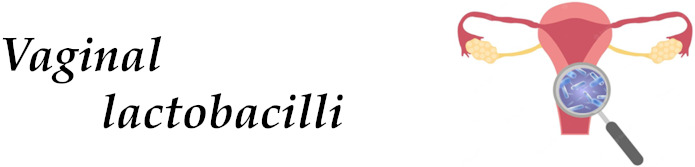
Lactic acid production
➢ low vaginal pH inhibit the growth of several other microorganisms
Hydrogene peroxide generation
➢ antimicrobial, antiinflammatory and signaling activity
Bacteriocin synthesis
➢ specific antimicrobial activity against certain microorganisms
Epithelial cell adhesion
➢ competitive inhibition of colonization by other microorganisms
S-layer protein expression
➢ promoting cell adhesion and anti-inflammatory activity
